# MTSC-Net: A Semi-Supervised Counting Network for Estimating the Number of Slash pine New Shoots

**DOI:** 10.34133/plantphenomics.0228

**Published:** 2024-08-28

**Authors:** Zhaoxu Zhang, Yanjie Li, Yue Cao, Yu Wang, Xuchao Guo, Xia Hao

**Affiliations:** ^1^ College of Information Science and Engineering, Shandong Agricultural University, Taian, 271018, Shandong Province, China.; ^2^ Research Institute of Subtropical Forestry, Chinese Academy of Forestry, Hangzhou 311400, Zhejiang Province, China.; ^3^ School of Big Data, Taishan College of Science and Technology, Taian, 271034, Shandong Province, China.

## Abstract

The new shoot density of slash pine serves as a vital indicator for assessing its growth and photosynthetic capacity, while the number of new shoots offers an intuitive reflection of this density. With deep learning methods becoming increasingly popular, automated counting of new shoots has greatly improved in recent years but is still limited by tedious and expensive data collection and labeling. To resolve these issues, this paper proposes a semi-supervised counting network (MTSC-Net) for estimating the number of slash pine new shoots. First, based on the mean-teacher framework, we introduce the improved VGG19 to extract multiscale new shoot features. Second, to connect local new shoot feature information with global channel features, attention feature fusion module is introduced to achieve effective feature fusion. Finally, the new shoot density map and density probability distribution are processed in a fine-grained manner through multiscale dilated convolution of the regression head and classification head. In addition, a masked image modeling strategy is introduced to encourage the contextual understanding of global new shoot features and improve the counting performance. The experimental results show that MTSC-Net outperforms other semi-supervised counting models with labeled percentages ranging from 5% to 50%. When the labeled percentage is 5%, the mean absolute error and root mean square error are 17.71 and 25.49, respectively. These findings demonstrate that our work can be used as an efficient semi-supervised counting method to provide automated support for tree breeding and genetic utilization.

## Introduction

Native to the southeastern United States, *Pinus elliottii*, also known as slash pine, grows in slash forests. Due to their rapid growth, drought resistance, waterlogging resistance, and high yield of turpentine, a wide range of these plants have been planted in southern China [1,2]. In recent years, with the growing role of slash pine in forestry planting, researchers have gradually integrated bioinformatics [3], high-throughput multispectral unmanned aerial vehicles [4], machine learning, and deep learning (DL) [5,6] to analyze the slash pine phenotype. Among the many phenotypic traits, the number of new shoots per slash pine represents the new shoot density, and it can be used as an essential indicator to measure wood selection and resin yield. The new shoot density is closely related to nutrient absorption, tree growth, and the volume of slash pine; it is also related to the photosynthetic ability of the tree crown [7]. However, manual counting is currently the most commonly used method for calculating new shoot density. Due to the terrain, tree volume, branch density, etc., this calculation method can be challenging because of the efficiency and accuracy of shoot counting and the safety of counting personnel.

With the rapid development of DL, revolutionary changes have taken place in the field of plant phenotyping. DL can effectively process massive amounts of high-dimensional big data and automatically learn to extract features [8], which helps extract rich information from complex plant phenotypic data. It has been widely used in plant detection [9], classification [10,11], segmentation [12,13], and other applications. DL has also been widely used to accurately count plant traits, such as wheat ear counting [14], maize tassel counting [15], slash pine new shoot counting [5], and soybean pod counting [16]. Methods based on density estimation are supervised through point labels and sum the targets from the density map via integration, and objects can be accurately located and counted in dense scenes via these methods [17–19]. However, the training of counting models requires large quantities of data with labeling and relies on tedious point labeling [20,21]. In previous work, we constructed a slash pine new shoot counting dataset (NSCD) and labeled 36,758 new shoots in 313 images, preliminarily implementing the new shoot counting task [5]; however, the normal implementation of the above work generally needs to be supported by sufficient and accurate data, and this process is not only time-consuming but also costly when it involves manual labeling. Therefore, ensuring the counting accuracy and robustness of the model while lowering the labeling cost is the key to improving the counting of slash pine new shoots. It is also an important research topic for the application and development of DL models.

In recent years, the combination of semi-supervised learning (SSL) and DL has been gradually applied in crowd counting tasks [21–26] (the application of SSL in crowd counting is discussed in detail in “Semi-supervised counting”). With the demand for agricultural trait counting, SSL also has good application prospects, as, for example, the dense distribution of grapes and the high labeling cost made counting very difficult. Fortunately, Li et al. pioneered a semi-supervised counting method for field grape berries named CDMENet. Compared with the current fully supervised and semi-supervised models, it results in superior counting performance with fewer labeled images [27]. Amirkolaee et al. proposed TreeFormer, which was the first tree counting method based on a semi-supervised transformer framework. When the amount of manual labeling was reduced by 70%, the accuracy was close to that of the fully supervised model [28]. Bogomasov et al. used an iterative semi-supervised training strategy for counting fruits and vegetables. By reducing the manual labeling workload by 60%, they conducted experiments on 36 different target categories and maintained 83% counting accuracy [29]. Furthermore, a new semi-supervised maize seedling leaf counting method was conducted by Xu et al.; specifically, by training SOLOv2 to segment maize seedlings, which achieved a mean average precision of 93.6% when the manual labeling cost was reduced by 70% (the labeled percentage was 30%), YOLOv5x was trained to count maize leaves. When the manual labeling cost was reduced by 60% (the labeled percentage was 40%), the segment and counting performance was comparable to that of fully supervised methods [30]. Compared with traditional fully supervised learning methods, SSL can utilize a small number of labeled data and a large number of unlabeled data for training, markedly reducing the workload and cost of data labeling and ensuring counting performance. Moreover, by combining labeled and unlabeled data, SSL can provide more comprehensive and diverse information for training models, which helps the model better understand the counting scenarios and features, promotes the generalizability and accuracy of the model, and makes the model more adaptable. Without the need for relabeling and training, it can handle counting tasks in new scenarios, which will also help expand its adaptability to new scenarios.

To the best of our knowledge, slash pine new shoots have not been counted via SSL at this time in other studies. Therefore, this paper proposes constructing the MTSC-Net for estimating the number of slash pine new shoots. This method counts new shoots at a low labeling cost and has good counting performance. First, the basic framework of MTSC-Net was based on the commonly used SSL framework—the mean-teacher framework [31]. To guide the student and teacher models to improve their understanding of new shoot counting scenarios, we chose a patch-aligned random masked image modeling (MIM) strategy [32] to process the dataset. Second, we captured the training scene context information by introducing the attention feature fusion (AFF) with a multiscale channel attention module (MS-CAM) [33]. The AFF can overcome the problems of semi-supervised counting models, which are prone to overreliance on the local information of the labeled data and thus overfitting the labeled dataset. Finally, efficient regression and classification heads with multiscale receptive fields [34] are crucial for performing fine-grained processing of density relationships and determining the geometric properties of new shoot features, improving the effectiveness with which changes are detected in the global scale and density. By designing loss functions for the student model and the teacher model separately, the student and the teacher could make full use of the feature relationship between labeled and unlabeled images, optimize the model, and improve the performance of new shoot counting.

As a result of this work, the following contributions have been made:

1. A semi-supervised counting method with the patch-aligned random masking strategy is proposed that uses a limited number of labeled data to count new shoots in complex backgrounds. This approach solves the counting problem of dense irregularly distributed images at a low labeling cost.

2. AFF and multiscale dilated convolution (MDC) modules are introduced to enhance the feature fusion ability and increase the accuracy, robustness, and generalization capabilities of the model when new shoots are counted.

3. The loss functions for different tasks, i.e., labeled and unlabeled datasets, are designed to promote mutual learning and the integration of student–teacher models.

### Crowd counting

Research on object counting has included a variety of types, but crowd counting has undergone the fastest development and the most related research. Therefore, this section mainly reviews the counting task on the basis of related research on crowd counting.

Currently, most counting research focuses on detection, regression, and density-based estimation. Methods based on detection predict each person’s bounding boxes, and the predicted results were determined by the number of boxes. For example, Liu et al. [35] proposed DecideNet to estimate the crowd density through density maps via detection and regression and adaptively evaluated the reliability of the 2 estimates through the attention module. Li et al. [18] proposed CSRNet for crowd recognition in crowded scenes, and it can efficiently predict crowds by generating density maps with accurate localization. Crowd counting frameworks based on density estimation have also been widely studied. The count result was obtained by accumulating the density map pixels, and the density map was generated pixel by pixel [18] and block by block [36]. Ma et al. [17] designed a loss function based on Bayesian theory for crowd counting. Optimized transport also improved the counting results when the predicted density map was compared with the true density map [19]. However, differences in scale changes, complex disturbance backgrounds, and cluster distribution heterogeneity have become major challenges in object and crowd counting tasks. Complex network architectures were often used as the backbone to obtain multiscale features to solve this challenge. Through reasonable feature fusion, the issue of counting crowds at large scales can be effectively solved, with examples including the multiscale feature fusion network (MFFNet) [37] with cascaded supervision and the contextual attention fusion network (CAFNet) [38]. Moreover, VGG16/19 also performed well in the counting field with the feature fusion module. PSGCNet combined VGG19, a pyramidal scale module and a global context module for efficient counting [39]. By combining VGG16, the multiscale feature extraction module and attention-based fusion module also achieved excellent counting performance [40]. MTSC-Net also used VGG19 as the feature extraction structure and introduced the AFF module to fuse multiscale context information and enhance the relationship between different position and angle features, thereby better addressing multiscale extraction. Thus, it could better handle the multiscale new shoot counting problem.

### Semi-supervised counting

In recent years, SSL based on consistency regularization has been the main type of SSL studied. Among them, most methods augment unlabeled data multiple times and encourage maintaining the consistency of augmented prediction results or results from different augmented views with the same input; the mean-teacher model [31] and the FixMatch model [41] are 2 main examples. Other methods, such as SSL with pseudo-labels, complete semi-supervised tasks by training on labeled data and making predictions on unlabeled data and use the prediction results as pseudo-labels to expand the training set to improve model performance, such as MixMatch [42] and ReMixMatch [43]. Moreover, MixMatch and ReMixMatch are also usually used as hybrid methods, combining regularization and pseudo-labeling, and have also been widely used in recent years.

The SSL-based crowd counting task attempts to achieve a counting performance that is comparable to fully supervised counting while reducing the labeling cost. For example, L2R [23] proposed a novel crowd counting method that employed a learning ranking framework. This approach simultaneously conducted image ranking and crowd density estimation via a multitask network. IRAST [26] used unlabeled images to achieve more reliable and efficient semi-supervised crowd counting by learning feature extractors and binary segmentation tasks. DACount [25] utilized SSL and integrated strategies such as density proxies, contrastive learning, transformer structure, and noise suppression to achieve efficient crowd counting. DREAM [44] leveraged a large number of unlabeled images and capitalized on the relationships among pyramid features to perform effective crowd counting, adhering to the deep ranking consistency principle. Our MTSC-Net was based on the mean-teacher framework of traditional consistent learning and improves the model performance and generalization abilities by allowing the student model to learn better through teacher supervision and guidance. This outcome was achieved by generating density maps and density probability distributions by constructing a regression head and classification head. As a result, the overall model performance and generalization ability improve.

## Materials and Methods

### Construction of the NSCD dataset

In our self-constructed NSCD dataset, the source of the photos of slash pines is located in the subtropical climate zone in China, specifically the Matou National Forest Farm in Xuancheng city, Anhui (30°45′N, 118°29′E). The annual precipitation at this site was 1,520 mm, and the average annual temperature was 15.7°C. The growing area of slash pine was approximately 49.4 acres, with a good growing environment and dense growth, providing a strong raw original dataset.

**Fig. 1.**
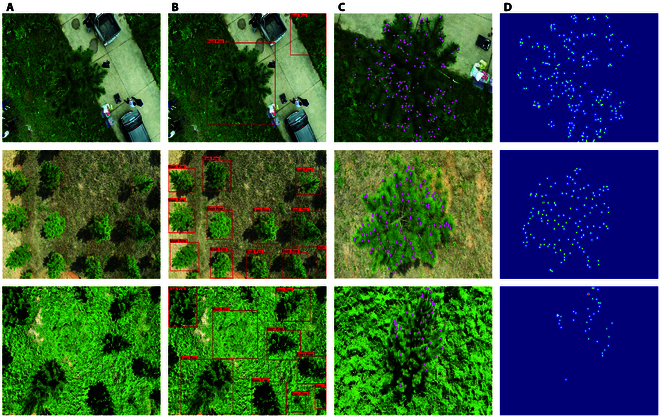
Examples of acquisition of the NSCD dataset. (A) Slash pine in different distribution backgrounds. (B) Individual slash pine extracts. (C) Labeling of new shoots. (D) Density map of new shoots.

The construction of the NSCD dataset was divided into 4 steps (Fig. 1). First, the data were collected by a DJI Phantom 4 RTK unmanned aerial vehicle (DJI, Shenzhen, Guangdong, China) equipped with a 4,864 × 3,648 resolution RGB camera. The acquisition time was set to March and June 2022 to collect images of slash pine at different growth stages. Cloudy days were chosen for drone photography, and direct sunlight was avoided when the drone flew. The overlap percentages of the side and front images of the NSCD dataset were 80% and 85%, respectively. To make the model more generalized, we selected different backgrounds of slash pines during the acquisition process, which can be divided into 3 types according to the different backgrounds distributed on roads, soil, and grass, as shown in Fig. 1A. Second, we extracted single slash pine trees through our previously published single slash pine extraction network, SPSC-net [5], as shown in Fig. 1B. A total of 313 images were extracted, each with a resolution of 1,024 × 768 dpi. The ground truth was subsequently obtained by manually labeling the NSCD dataset, as shown in Fig. 1C. Due to the low resolution of the images, a total of 2 months of manual labeling time were used during the labeling process, and the number of slash pine new shoots in each image ranged from 80 to 246. A total of 36,758 new shoots were labeled in 313 images. Finally, MTSC-Net adopted the geometric adaptive kernel method described by Li et al. [18] to process the NSCD dataset, and the labeled new shoot point coordinates were mapped to the density map. Using a density map for supervision when training the labeled data, the irregularly distributed slash pine new shoots are shown in the highlighted part of Fig. 1D. The NSCD dataset was divided at a ratio of 7:2:1, and the 3 subsets were used for model training, validation, and result testing. To ensure the rationality of the experiment, we randomly generated images with labeling from 5% to 50% of the labeled percentages from the training set to verify the learning ability of the model with only a small number of labeled data and a large number of unlabeled data and to prove the efficiency of the model.

### Problem definition

We divided the NSCD dataset into a labeled dataset *L* and an unlabeled dataset *U*. $L={\left\{\left(I_{i}^{l},D_{i}^{\;\mathrm{gt}}\right)\right\}}_{i=1}^{N_{l}}$ and *N_l_* represents the number of labeled images, expressed as $I_{i}^{l}$, respectively, and $D_{i}^{\;\mathrm{gt}}$ represents the manually labeled density map. $U={\left\{I_{i}^{u}\right\}}_{i=1}^{N_{u}}$ represents the unlabeled dataset, where *N_u_* represents the number of unlabeled images and where $I_{i}^{u}$ represents each unlabeled image, which is not a corresponding density map. In addition, in the NSCD dataset, *N_u_* ≫ *N_l_*. Through MTSC-Net, we aim to perform better than training only on *N_l_* by training only on *N_u_*, thereby obtaining a new shoot counting model with fewer labeling costs but a better counting effect.

### Overall design of MTSC-Net

**Fig. 2.**
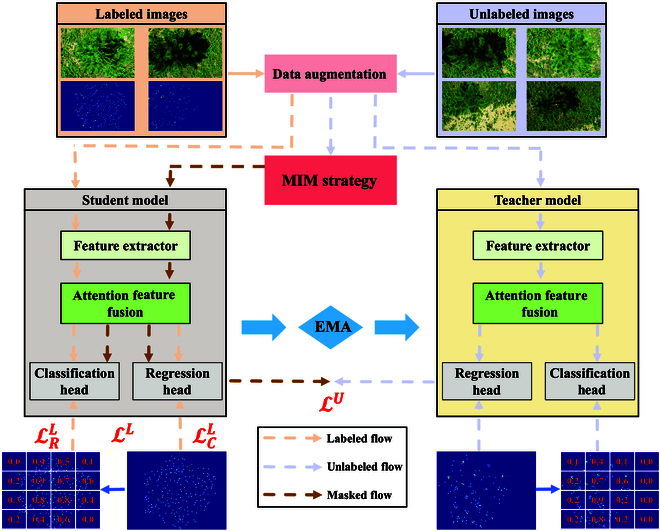
Detailed structure of MTSC-Net.

The overall structure of MTSC-Net is illustrated in Fig. 2, which contains a left-branch student model and a right-branch teacher model. The models have the same backbone network and use the expotential moving average (EMA) weights to update the teacher model. The model network includes a backbone network for extracting multiscale features, an AFF module, a regression head, and a classification head with the MDC. Through the feature extraction structure and fusion and after the final fine-grained processing, the model finally predicts density maps, and density probability distributions adjust and optimize the model. Next, these module processes are described respectively.

### Feature extraction

**Fig. 3.**
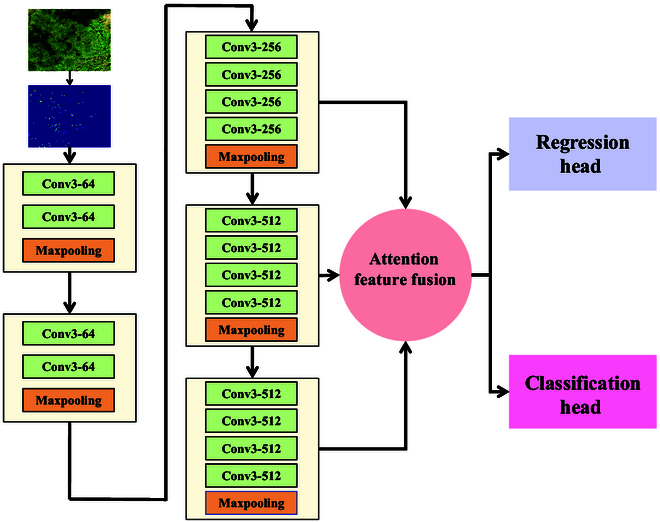
Feature extraction network structure.

In this section, we present the basic backbone network for feature extraction of slash pine shoots, and we present an improved VGG19 schematic that can capture more detailed deep features from densely distributed new shoots. The input sample size is ( *H* × *W* × 3), and images with deep features are extracted through the first 5 convolutional layers. The last 3 convolutional layers (sizes are $\frac{H}{8}\times \frac{W}{8}\times 256$, $\frac{H}{8}\times \frac{W}{8}\times 512$, and $\frac{H}{8}\times \frac{W}{8}\times 512$) are used for multiscale AFF, and finally, the features flow to the regression head and classification head. The feature extraction structure is shown in Fig. 3.

### Masking strategy

To promote the contextual understanding of the model in terms of the extracted new shoot features, we consider fine-grained processing of the NSCD dataset. The inspiration for this section came from Zeng et al. and Qian et al. [45,46], and the MIM [32] strategy was introduced to perform patch-based random masking on unlabeled images to improve the learning capability of the model. In this way, the semantic and structural features of the image are learned by masking some areas in the image and predicting missing pixels so that the model can better understand and express new shoot distributions and density information in the image. In addition, we combined brightness changes and flip scaling to avoid counting models that rely on detailed features such as new shoot size and color.

**Fig. 4.**
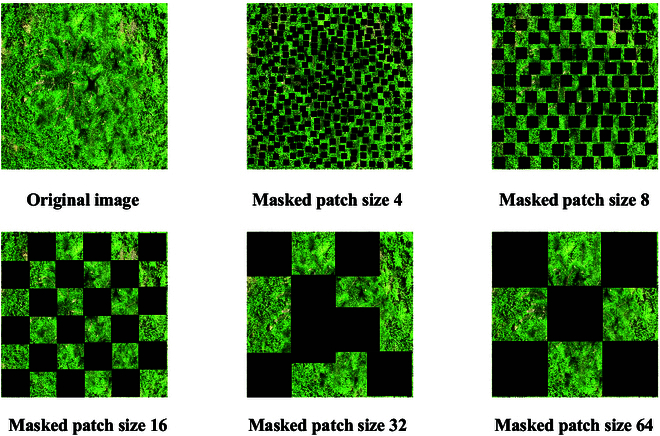
Slash pine images processed via the MIM strategy.

Specifically, our student model learns new shoot features through labeled images and masked unlabeled images. The student also extracts new shoot clues around the masked image block from unlabeled images so that it can better model the contextual relationship of the new shoot counting scene to enhance the understanding of feature learning and infer and predict the masked area. However, in fully supervised learning, counting models usually learn contextually through the ground truth as supervision, whereas in MTSC-Net, to promote better model learning and mutual communication, we chose the predicted values from the teacher model as supervision. The teacher model makes predictions on unlabeled images and provides supervision signals to the student model, thereby effectively supervising and guiding the training of the student model, communicating and merging through the EMA. During this process, we emphasized the independent acquisition of filled masked areas by the student model and encouraged improvements in the ability to predict masked new shoots through known information and contextual relationships. This approach helps improve the prediction performance of MTSC-Net for low-density areas while combining local details to predict high-density areas. Figure 4 shows the image of slash pine processed by the MIM.

### Attention feature fusion

**Fig. 5.**
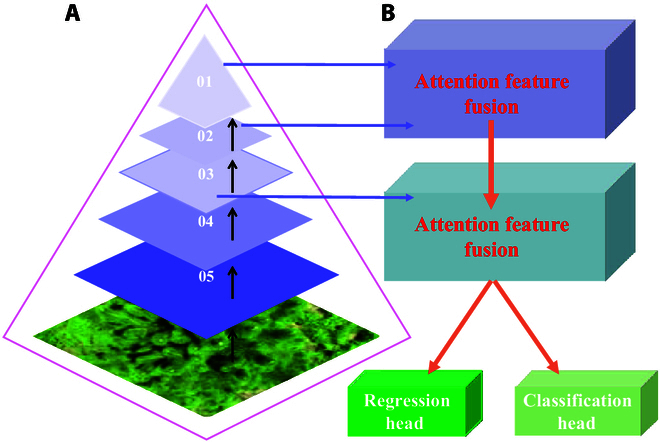
Usage of the AFF in MTSC-Net. (A) Feature extraction. (B) Features processed by the AFF.

**Fig. 6.**
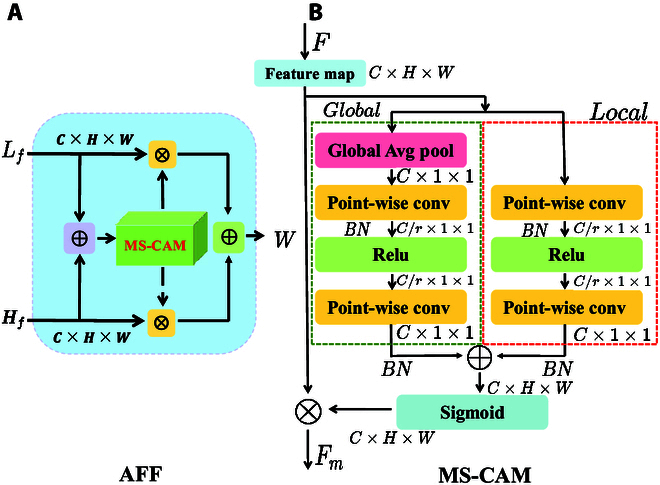
Detailed architecture of AFF module and MS-CAM module. (A) AFF module. (B) MS-CAM module.

To improve the learning ability of MTSC-Net for global slash pine new shoot features, the AFF module [33] was introduced after the improved VGG19 network. This module can precisely address the problem of the irregular distribution of small new shoot information and effectively capture the spatial distribution information and dependence of new shoot features, as shown in Fig. 5. In general, the plug-and-play attention mechanism module helps to enhance the ability of feature learning, but it focuses only on features at a certain level and cannot better integrate feature relationships between different levels in the new shoot counting scenario. In our previously published software, CountShoots [5], by combining high- and low-level new shoot features, the pyramid feature aggregation model achieved a good counting effect. However, in a follow-up study, we found that establishing the feature relationship between extracted features of different scales and kernels only by addition, although increased feature utilization, could not focus on effective feature screening. Therefore, we introduced the feature recognition module AFF, which can focus on extreme scale changes by paying attention to both spatial attention and channel attention. The AFF can effectively fuse features at a low level *L_f_* (i.e., local location shoot information) and features at a high level *H_f_* (i.e., global channel shoot feature), thereby fusing multiscale contextual information and selecting effective new shoot features. By using MS-CAM in the AFF, attention weights *M* ∈ [0 , 1] are obtained. Figure 6A shows the structure of the AFF, and the obtained feature map can be calculated as follows:

$$W=M\left(L_{f}\right.⊕\left.H_{f}\right)⊗L_{f}+\left(1-\left.M\left(L_{f}⊕H_{f}\right)\right)\right.⊗H_{f}$$ (1)

where *W* ∈ *R*^*C*×*H*×*W*^ denotes a new feature map. *C*, *H*, and *W* represent channels, the height, and the width, respectively. ⊕ denotes the addition operation, and ⊗ denotes the multiplication operation.

The key point of the AFF implementation is the addition of the MS-CAM module, whose structure is shown in Fig. 6B. To capture local location new shoot feature information, MS-CAM uses pointwise convolution (PWConv) to achieve local position feature fusion through point-by-point channel interaction. This approach can emphasize the local characteristics of small new shoots more strongly. *L*(*F*) is the local feature attention and is calculated as follows:

$$L\left(F\right)=\text{PWConv}\left(\text{Relu}\left(\text{PWConv}\left(F\right)\right)\right)$$ (2)

To achieve global feature attention *G*(*F*), MS-CAM also uses PWConv as the feature fusion convolution and matches it with the global average pooling (GAP) branch to pay attention to the global feature information. *G*(*F*) is calculated as follows:

$$\begin{array}{c} \mathrm{GAP}=\frac{1}{H\times W}\sum_{i=1}^{H}\sum_{i=1}^{W}F\left(H\;,\;W\right) \end{array}$$ (3)

$$G\left(F\right)=\text{PWConv}\left[\text{Relu}\left(\text{PWConv}\left(\mathrm{GAP}\left(F\right)\right)\right)\right]$$ (4)

where *Relu* represents the activation function. (*H*, *W*) represents the coordinates, and *F* denotes the pixel value of the point. The new shoot feature extracted via MS-CAM is calculated as follows:

$$F_{m}=F⊗M\left(F\right)=F⊗\left(\sigma \left(G\left(F\right)⊕L\left(F\right)\right)\right)$$ (5)

### Regression and classification head

**Fig. 7.**
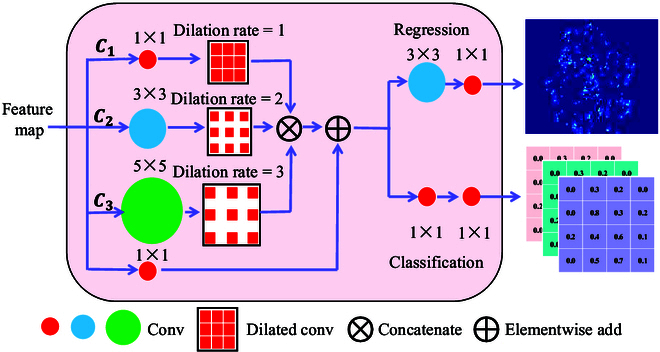
Regression head and classification head with the MDC.

Although the model is able to capture sufficient global information of the new shoots through the improved VGG19 backbone network and AFF module, the detail processing and feature learning ability generalization of the model are critical for the overall shoot feature learning. Therefore, MTSC-Net contributed to the success of model leaning adjustment by introducing the regression of accurate density maps and density probability distributions. We built the regression head and classification head by introducing the lightweight MDC [34] and by detecting global scale and density changes, density maps, and density probability distributions were efficiently generated. Its structure is shown in Fig. 7.

**Fig. 8.**
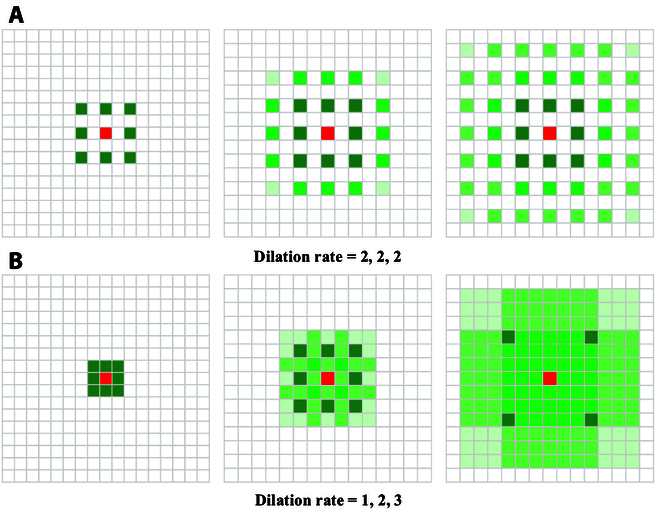
Differences in dilation rates when stacking DConv layers. (A) Effect of fixed dilation rate. (B) Effect of multiscale dilation rate.

A substantial difference between the MDC and stacked fixed dilation convolution (DConv) is that the MDC stacks DConv layers with different dilations in parallel. Specifically, as shown in Fig. 8, the dilation coefficient is carefully designed after each layer to prevent missing some pixels in subsequent convolutions while enlarging the receptive field. This approach can maintain the spatial resolution while enlarging the receptive field. The output feature maps of 3 columns (*C*_1_, *C*_2_, and *C*_3_) are concatenated and added to the feature maps of the shortcut paths to capture multiscale features. Finally, we regress density map by combining 3 × 3 and 1 × 1 convolution layers and use two 1 × 1 convolution layers to complete the probability distribution. By setting a smaller convolution kernel size and dilation rate, the model can better adapt to counting scenes full of small-scale new shoots.

### Design of the MTSC-net loss

Our student model learned through a limited number of labeled data, and the loss function obtained by training on labeled samples is recorded as $L^{L}$. It consists of $L_{R}^{L}$ and $L_{C}^{L}$. Among them, our calculation of loss $L_{R}^{L}$ is generalized to the losses studied by Rong et al. [47] and Qian et al. [48]. $L_{R}^{L}$ is used to generate loss for the prediction of the regression head, which improves the accuracy of overall prediction in densely distributed new shoot areas through prior knowledge. $L_{C}^{L}$ is used to generate the loss for the density probability distribution of the classification head. First, the calculation formula of $L_{R}^{L}$ is expressed as follows:

$$M^{\mathrm{gt}}=l\left(D^{\mathrm{gt}}>\varepsilon \right)$$ (6)

*M^gt^* is generated through the density map *D^gt^* obtained from manually labeled training samples. It is a binary segmentation map generated based on the threshold *ε* to distinguish dense areas and sparse areas. It can be obtained via the indicator function $l\left(D^{\mathrm{gt}}>\varepsilon \right)$, where here, *ε* is set to 1e-3.

$$\mathrm{DM}=D^{P}⊙M^{\mathrm{gt}}$$ (7)

$$\hat{\mathrm{DM}}=D^{\mathrm{gt}}⊙M^{\mathrm{gt}}$$ (8)

where *D^P^* represents the predicted density map. The symbol ⊙ denotes Hadamard multiplication. *DM* denotes the processed density map predicted through supervision, and $\hat{\mathrm{DM}}$ denotes the processed density map obtained from point labeling.

$$\text{SSIM}\left(X\right.,\left.Y\right)=1-\left(\frac{\left(2\mu_{X}\mu_{Y}+Z_{1}\right)\left(2\sigma_{\mathrm{XY}}+Z_{2}\right)}{\left(\mu_{X}^{2}+\mu_{Y}^{2}+Z_{1}\right)\left(\sigma_{X}^{2}+\sigma_{Y}^{2}+Z_{2}\right)}\right)$$ (9)

where *SSIM* represents the structural similarity index measure [49].*SSIM* individuals utilizing high-quality density maps can concentrate on spatial intricacies and enhance structural resemblance. *μ* represents the local mean values used to calculate *μ_X_* and *μ_Y_*, and *σ_XY_* denotes the local covariance. The local variances of *X* and *Y* are expressed as $\sigma_{X}^{2}$ and $\sigma_{Y}^{2}$, respectively, and *Z*_1_ and *Z*_2_ represent constants, which are set to 0.01 and 0.03, respectively.

$$\mathrm{SL}=\frac{1}{Κ}\sum_{j=1}^{K}\left(1-\text{SSIM}\left({\text{Pool}}_{j}\left(\mathrm{DM}\right),{\text{Pool}}_{j}\left(\hat{\mathrm{DM}}\right)\right)\right)$$ (10)

$$L_{R}^{L}=\mathrm{SL}+\lambda_{1}L_{\mathrm{TV}}\left(D^{P}\right.,\left.D^{\mathrm{gt}}\right)$$ (11)

where SL represents the structural loss [47], which downsamples the image by *Pool_j_* to size $\frac{1}{2^{\;j-1}}$. The value of *K* is 3. $L_{\mathrm{TV}}$ is the total variation loss [19], and *λ*_1_ is the weight factor, which is set to 0.01. Additionally, $L_{C}^{L}$ is calculated as follows:

$$L_{C}^{L}=\frac{1}{N_{l}}\sum_{j=1}^{N_{l}}H\left(y_{j}^{\mathrm{gt}}\right.,\left.y_{j}^{\;p}\right)$$ (12)

For new shoot counting, we treat the small new shoot density distribution as a class and perform a loss calculation on the model predictions versus the ground truth via the cross-entropy function H. $y_{j}^{\mathrm{gt}}$ represents the ground truth of the new shoot density probability distribution, and $y_{j}^{\;\;p}$ represents the predicted new shoot density probability distribution. By combining H with the *softmax* activation function, the output of the new shoot counting model is converted into a class probability distribution, which can better distinguish different new shoot features. By maximizing mutual information, the accuracy of the new shoot counting is improved. The loss $L^{L}$ can be summarized as follows:

$$L^{L}=L_{R}^{L}+L_{C}^{L}$$ (13)

Our student and teacher communicated and optimized each other by predicting unlabeled training samples with and without masking. Better encourage student models to learn global new shoots features. The unsupervised loss $L^{U}$ also consists of $L_{R}^{U}$ for density regression and $L_{C}^{U}$ for density probability distribution and is calculated as follows:

$$L_{R}^{U}=\frac{1}{N_{u}}\sum_{j=1}^{N_{u}}\sum_{i\in \Omega }L_{1}\left({\hat{D}}_{\mathrm{ij}}^{s}\right.,\left.{\hat{D}}_{\mathrm{ij}}^{t}\right)$$ (14)

$$L_{C}^{U}=\frac{1}{N_{u}}\sum_{j=1}^{N_{u}}\sum_{i\in \Omega }L_{1}\left({\hat{y}}_{\mathrm{ij}}^{s}\right.,\left.{\hat{y}}_{\mathrm{ij}}^{t}\right)$$ (15)

$$L^{U}=L_{R}^{U}+L_{C}^{U}$$ (16)

where *Ω* represents the number of masked patches per unlabeled image, and ${\hat{D}}_{\mathrm{ij}}^{s}$ and ${\hat{y}}_{\mathrm{ij}}^{s}$ represent the new shoot density map and density probability distribution derived from the student predictions, respectively. The values predicted by the teacher model are treated as the ground truth, which are ${\hat{D}}_{\mathrm{ij}}^{t}$ and ${\hat{y}}_{\mathrm{ij}}^{t}$. Furthermore, when the smoothed $L_{1}$ is used, the network becomes more robust. Through $L^{L}$ and $L^{U}$, the overall loss of MTSC-Net is summarized and calculated as follows:

$$L=L^{L}+L^{U}$$ (17)

Algorithm 1 illustrates the training process of MTSC-Net as follows:


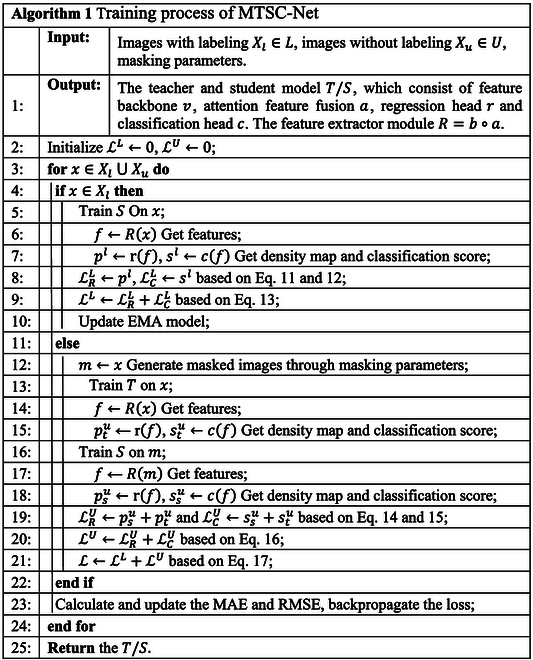


## Results

### Experimental details

All of our experiments were completed in the same training environment. Some details of the training server include an Intel(R) Core(TM) i9--12900K CPU and an NVIDIA GeForce RTX 3090 graphics card. The operating system was Windows 10, the RAM size was 64 GB, and PyTorch 1.13.1 was used. All code was run in the PyCharm 2022.3.1 software, in which the CUDA version was 11.6. The masked patch size was set to 16, and the masking ratio was set to 0.1. The crop size was 256 × 256, and the batch size was 16. The number of epochs was 500. The feature maps of the last 3 different levels were extracted through the backbone, and the output channels were 256, 512, and 512, respectively. The last 2 features with an output channel of 512 were fused through the AFF and then fused with the feature map with an output channel of 256 through the AFF.

### Evaluation metrics

At present, most counting models use the mean absolute error (MAE) and root mean square error (RMSE) as evaluation indicators. The MAE is used to reflect the deviation between the predicted value obtained by the model and the ground truth, while the RMSE can provide a more accurate measurement of the prediction error and is very sensitive to the outliers generated by the model. MTSC-Net also uses these 2 methods as evaluation indicators for the new shoot counting model, which are calculated as follows:

$$\mathrm{MAE}=\frac{1}{N}\sum_{i=1}^{N}\left|P_{i}-G_{i}\right|$$ (18)

$$\text{RMSE}=\sqrt{\frac{1}{N}\sum_{i=1}^{N}{\left|P_{i}-G_{i}\right|}^{2}}$$ (19)

where *N* denotes the slash pine samples in the test set from the NSCD dataset. The *i*th image is represented by *P_i_* and *G_i_*, which are the predicted and actual numbers of new shoots, respectively.

### Counting performance of MTSC-Net

This section mainly verifies the efficiency of MTSC-Net in terms of slash pine new shoot counting. An analysis of the NSCD dataset was conducted to compare it with other counting models, including the fully supervised counting models, i.e., MCNN [50], CSRNet [18], DM-Count [19], SPSC-net [5], and semi-supervised counting models, i.e., Dream [44], Calibrating [22], DACount [25], MRC [46], and MTCP [24]. The values in Table 1 represent the MAE and RMSE values of different fully supervised models on the test set.

**Table 1.** A comparison of different fully supervised models for counting new shoots

Model	MAE	RMSE
MCNN	30.76	31.12
CSR-net	6.50	27.06
DM-Count	6.84	12.85
SPSC-net	2.27	7.00

As shown in Tables 1 and 2, even though MTSC-Net uses a semi-supervised counting method, when MTSC-Net uses 5% labeled data, the number of images is only 10. There are far fewer labeled images than unlabeled images, but MTSC-Net yields MAE and RMSE values of 17.71 and 25.49, respectively. By dramatically reducing manual labeling costs, compared with the fully supervised model CSRNet, the RMSE of MTSC-Net is still reduced by 5.80%. Compared with the MCNN, there is a great reduction of 42.43% in the MAE and a reduction of 18.09% in the RMSE. When the labeled percentage is 50%, compared with the fully supervised algorithms DM-Count and SPSC-net, MTSC-Net also provides better new shoot counting results with lower labeling costs. The differences between MAE for DM-Count and SPSC-net are only 1.94 and 6.51, respectively.

**Table 2.** Different semi-supervised models for the counting of new shoots are compared

Labeled percentage	Model	MAE	RMSE
5%	Dream	103.63	135.48
	Calibrating	37.28	47.32
	MTCP	32.74	44.13
	DACount	21.45	30.31
	MRC	19.48	27.95
	MTSC-Net(ours)	17.71	25.49
10%	Dream	81.97	99.11
	Calibrating	31.44	39.64
	MTCP	29.41	37.80
	DACount	20.51	26.48
	MRC	18.77	24.19
	MTSC-Net(ours)	16.24	20.68
20%	Dream	44.62	51.40
	Calibrating	26.14	30.78
	MTCP	22.59	28.35
	DACount	20.24	26.77
	MRC	15.35	21.68
	MTSC-Net(ours)	11.28	17.30
30%	Dream	26.42	33.52
	Calibrating	24.56	30.40
	MTCP	19.75	27.62
	DACount	17.67	24.28
	MRC	13.79	18.78
	MTSC-Net(ours)	10.20	17.98
40%	Dream	23.93	31.38
	Calibrating	21.88	28.02
	MTCP	16.55	21.23
	DACount	16.93	22.04
	MRC	12.21	17.32
	MTSC-Net(ours)	9.73	16.64
50%	Dream	19.91	25.42
	Calibrating	20.91	27.34
	MTCP	14.42	18.08
	DACount	8.91	17.62
	MRC	10.48	15.69
	MTSC-Net(ours)	8.78	16.60

**Fig. 9.**
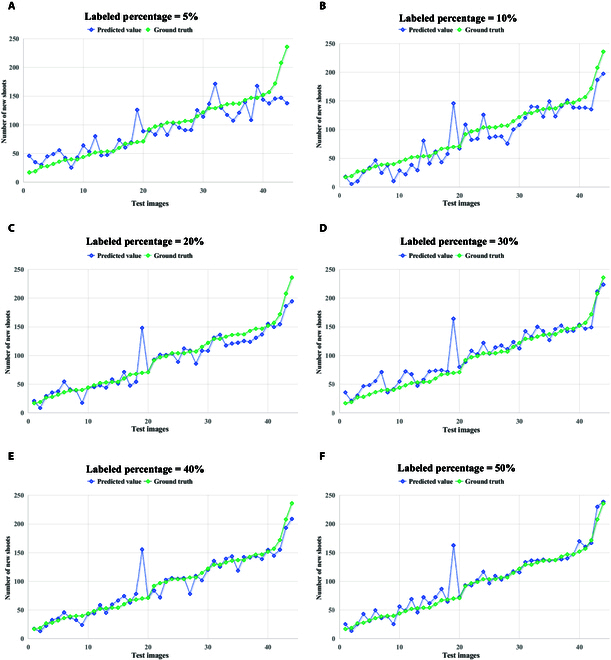
Comparison between the ground truth and predicted values at different labeled percentages. (A to F) The x-axis represents each test image, and the y-axis represents the number of new shoots in the image.

We can analyze from Table 2 that different semi-supervised models performed differently on the test set in terms of the MAE and RMSE, and MTSC-Net achieves fewer counting errors at different percentages. Notably, when the labeled percentage is 20% and the number of labeled data is 40, compared with the better-performing MRC algorithm, MTSC-Net reduces the MAE and RMSE by 26.51% and 20.20%, respectively. Figure 9 shows the relationship between the predicted values and ground truth of MTSC-Net for different percentages of labeled datasets. It is worth considering that the quality of the dataset and random allocation have a very large impact on MTSC-Net. As the number of labeled data increases, the increases in the MAE and RMSE are also very obvious. However, due to the low resolution of our dataset, the decrease in the RMSE is not very obvious during the process of gradually increasing the dataset labeled percentages by 30% to 50%. This observation further proves that the quality of labeled data is critical for model training and further improvement. Our next step is to continue optimizing the model and to explore in depth how to enhance the ability of MTSC-Net to capture features and count generalizability when the image resolution is low.

### Evaluation of the AFF and MDC

To validate the importance and effectiveness of the AFF and MDC in MTSC-Net, this experiment stipulates that the labeled percentage is uniformly 50%. We selected VGG19 as the basic feature extractor and conducted ablation experiments while the parameters of each model remained consistent. When validating the effectiveness of the MDC, we used general parameter settings, changed the regression head to two 3 × 3 convolution layers and a 1 × 1 convolution layer, and changed the classification head to 2 layers of convolution layers with a kernel size of 1. The results of the ablation experiment are shown in Table 3.

**Table 3.** Results of ablation experiments with different modules

Models	AFF	MDC	MAE	RMSE
1			63.92	78.25
2		√	48.54	63.07
3	√		12.32	18.36
4	√	√	8.78	16.60

After adding the AFF, compared with Model 1 without feature fusion and the MDC, the MAE is reduced by 80.73%, and the RMSE is reduced by 76.54%. After adding the AFF, the ability to capture new shoot features is markedly improved. Model 4 shows that after we introduced the AFF and MDC at the same time, the MAE and RMSE were reduced by 86.26% and 78.79%, respectively. It is proved that after adding the AFF module and MDC module, by increasing the receptive field of the regression head and classification head, MTSC-Net can better fuse the feature information between different density levels and improve the potential of the model to explore the attention context of unlabeled images.

### Evaluation of the masking strategy

**Fig. 10.**
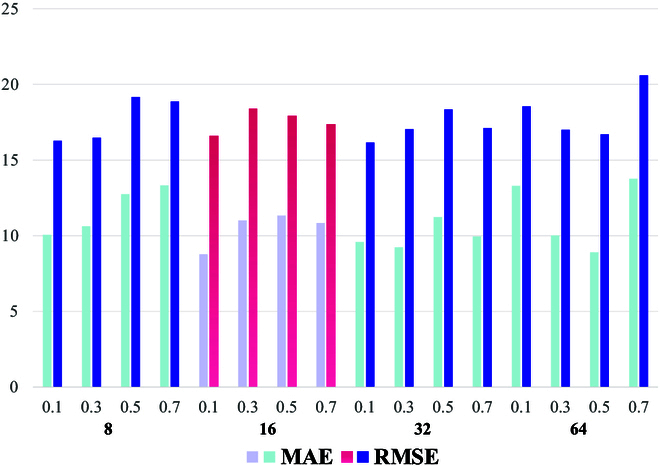
The impact of different masked patch sizes and masking ratios on MTSC-Net.

The MIM strategy consists of 2 parts. The masked patch size represents the block size of the masked image. In our experiments, we explored the impacts of 8, 16, 32, and 64 on MTSC-Net. The masking ratio represents the proportion of randomly distributed mask blocks. We explored the impacts of 0.1, 0.3, 0.5, and 0.7 on MTSC-Net. To validate the impact of the 2 MTSC-Net parameters, we set the labeled percentage to 50% and conducted an ablation experiment to compare the new shoot counting performance. As seen from the experimental results in Table 4, when the masked patch size is 16 and the masking ratio is 0.1, MTSC-Net has the best effect. At this time, the lowest MAE is 8.78, the RMSE has also been reduced to 16.60, and the overall error is the lowest. In addition, an analysis revealed that changes in masked patch size and masking ratio have little effect on the performance of MTSC-Net (Fig. 10), confirming its stability and robustness. For other labeled percentages, we also set the masked patch size and masking ratio to 16 and 0.1, respectively.

**Table 4.** The impact of different masking strategies selected by MTSC-Net on new shoot counting

Masked patch size	Masking ratio	MAE	RMSE
8	0.1	10.06	16.27
	0.3	10.63	16.48
	0.5	12.75	19.17
	0.7	13.32	18.86
16	0.1	8.78	16.60
	0.3	11.01	18.37
	0.5	11.33	17.93
	0.7	10.84	17.35
32	0.1	9.59	16.15
	0.3	9.24	17.04
	0.5	11.24	18.34
	0.7	9.95	17.11
64	0.1	13.29	18.55
	0.3	10.02	17.00
	0.5	8.91	16.70
	0.7	13.76	20.60

## Discussion

The effectiveness of MTSC-Net can be discussed from 3 perspectives:

1. MTSC-Net achieves satisfactory results: The AFF module can effectively fuse the global-level “abstract” and local-level “detailed”multiscale features, and the regression head and classification head solve the underlying flow pattern feature learning problem of the new shoot distribution. Furthermore, models can complete learning modeling through the density relationship between different regions in the multiscale density map. In addition, by introducing the MIM strategy, the model is encouraged to obtain clues independently to make predictions in masked patches of unlabeled images, improving the new shoot counting performance.

2. The reliability and effectiveness of the AFF and rationality of the fusion location: Although feature fusion is prevalent in counting networks, multiscale feature fusion between different levels is achieved by combining kernels of different sizes, groups, or layers at different stages. However, complex paths are constructed, and the feature fusion achieved by simple addition or concatenation operations cannot determine whether it is suitable for small-sized objects, such as new shoot features. Additionally, certain attention mechanisms solely produce fusion weights through the global channel attention mechanism, which cannot target densely distributed new shoot features but is more suitable for dispersing broader features. However, for small objects such as new shoots, the receptive field of the predictor cannot match the scale range of the new shoot, thus weakening the feature of the new shoot. Therefore, selecting an appropriate attention fusion module is crucial for extracting new shoot features. The AFF presents MS-CAM as a solution to the issue of inconsistent features across various scales in AFF. By paying attention to both spatial attention and channel attention simultaneously, it can focus on both large, widely distributed objects and small, locally distributed objects and finally aggregate multiscale contextual features along the channel dimension. This approach helps identify new shoot features under extreme scale changes. The location of the fused features in MTSC-Net can capture feature representations at different abstraction levels. In VGG19, as the network depth increases, the abstractness of the feature map gradually increases, and the feature maps of higher layers are more sensitive to the boundaries and shapes of small new shoots. Compared with shallow networks, feature maps of higher layers can extract more abstract and obvious features and learn more accurate and semantically rich feature representations [51]. When pyramids are used for feature fusion, more high-level semantics and global new shoot features are needed; however, the shallow network mainly focuses on low-level local features, whereas the later layers gradually focus on higher-level semantics and global features. By fusing these features, the contextual learning ability of the model can be enhanced, making the model more focused on discriminative shoot features [52]. Moreover, MS-CAM in the AFF aggregates the local channel context, and the results of the experiments show that the addition of high-level feature fusion helps the network focus on the recognition of small target shoot through high-level feature fusion; however, as the network depth increases, the parameter cost also increases greatly [33]. By fusing the output of layers with strong feature extraction abilities at the cost of a more reasonable number of parameters, the shallow and deep semantic feature is comprehensively given to provide a more comprehensive and rich expression of the new shoot feature.

3. The introduction of the MDC module is crucial to MTSC-Net: To capture sufficient new shoot features, designing an efficient regression head and classification head are critical for MTSC-Net to automatically and accurately detect global new shoot density scale changes. Directly stacked DConv layers are prone to grid effects [53]; that is, some pixels are lost in subsequent convolutions, as shown in Fig. 8. This phenomenon especially affects the accuracy of the counting results of slash pine new shoots. When processing the captured feature maps through the regression head and classification head, due to the small scale of the new shoot, the loss of one pixel value disregards multiple new shoots, resulting in inaccurate counting results. The MDC achieves a close fit between convolutions by setting different dilation rates, preventing the loss of pixel values and capturing multiscale receptive fields, ultimately ensuring accurate counting of new shoots.

## Conclusion

To achieve accurate counting of slash pine new shoots in complex environments with limited labeling data and at a low labeling cost, this paper proposes a semi-supervised counting network (MTSC-Net). MTSC-net uses the mean-teacher model as the basic skeleton and introduces the AFF module to fuse multiscale contextual information and select effective features. More detailed density information is obtained by combining local position new shoot feature information with new shoot features from the global channel. A regression head and a classification head consisting of the MDC are added to enlarge the receptive fields, and density maps and density distributions are used to supplement the global feature information. In addition, we introduce the MIM strategy to encourage the contextual understanding of the model in terms of new shoot features and improve the counting performance of MTSC-Net. The performance of MTSC-Net is compared against that of both fully supervised and semi-supervised counting models. The experimental results reveal that MTSC-Net achieves a lower error rate than both algorithms do. When the labeled percentage is 5%, the MAE and RMSE are 17.71 and 25.49, respectively. When the labeled percentage is 50%, the MAE and RMSE are 8.78 and 16.60, respectively. To the best of our knowledge, this paper is the first to use SSL to count slash pine new shoots. By acquiring and processing features in a fine-grained manner, the model achieves better counting performance and results at a lower cost, providing better research support for forestry researchers. We consider analyzing the new shoot feature representation and counting mode through multimodal data fusion in future work, improving the new shoot counting efficiency through further research and providing reliable technical support for subsequent related counting research in other forestry fields.

## Data Availability

The model code and data used in this paper are publicly available at https://github.com/haohuihui5019/MTSC-Net-main.
